# Evidence of Hepatitis A Virus Person-to-Person Transmission in Household Outbreaks

**DOI:** 10.1371/journal.pone.0102925

**Published:** 2014-07-22

**Authors:** Lyana Rodrigues Lima, Adilson José De Almeida, Renata dos Santos Tourinho, Bárbara Hasselmann, Lia Laura Lewis Ximenez, Vanessa Salete De Paula

**Affiliations:** 1 Laboratório de Desenvolvimento Tecnológico em Virologia, Instituto Oswaldo Cruz, Fundação Oswaldo Cruz, Rio de Janeiro, Brasil; 2 Ambulatório de Hepatites Virais, Instituto Oswaldo Cruz, Fundação Oswaldo Cruz, Rio de Janeiro, Brasil; 3 Hospital Universitário Gaffrée e Guinle, Setor de Hematologia, Universidade Federal do Estado do Rio de Janeiro, Rio de Janeiro, Brasil; Centers for Disease Control and Prevention, United States of America

## Abstract

The person-to-person transmission of the hepatitis A virus primarily occurs in enclosed spaces, particularly in the presence of inadequate hygiene conditions and a high proportion of susceptible individuals. Thus, intimate family contact stands out as a risk factor for HAV infection dissemination. The present study aimed to evaluate the occurrence of household HAV transmission. Blood samples were collected from patients with hepatitis A (index cases) and their family members (contacts) that were referred to an ambulatory care clinic specializing in viral hepatitis. A total of 97 samples were collected from 30 families with a confirmed hepatitis A case (index case). Serological and molecular techniques for the diagnosis of hepatitis A were conducted on all samples. HAV infection (anti-HAV IgM + and/or HAV RNA +) was detected in 34.3% (23/67) of the contacts; 34.3% (23/67) of the contacts were immune to HAV, and 31.4% (21/67) were susceptible. In the household contacts, HAV immunity was significantly associated with older age; susceptibility to infection and HAV infection were associated with younger age. Household outbreaks were detected in 16/30 families studied. Co-circulation of subgenotypes IA and IB was found in the household outbreaks, and person-to-person transmission was evidenced in six of the household outbreaks, with 100% homology between the index case and contact strains. The results demonstrated the relevance of HAV household transmission, reaffirming the need for hepatitis A vaccine administration in susceptible contacts and effective infection control procedures to prevent the extension of household outbreaks.

## Introduction

Hepatitis A is an inflammatory liver disease that annually affects approximately 1.4 million individuals worldwide [1]. The disease is transmitted primarily via the fecal-oral route, and its incidence rate is strongly correlated with socioeconomic conditions and access to safe drinking water [2], [3]. Brazil currently has an intermediate incidence rate of hepatitis A, affecting approximately 7,000 people per year [4], the decline in the country's hepatitis A incidence has been attributed to recent improvements in sanitation and environmental planning. As a result of the decreased incidence, the number of susceptible adolescents and adults has increased, leading to a higher risk of outbreaks in these age groups where the disease tends to be symptomatic [5], [6]. Although hepatitis A has great impact on the Brazilian health, does not exist yet a program of routine childhood vaccination in the country.

The hepatitis A virus (HAV) is a *Hepatovirus* member of the *Picornaviridae* family and has a positive single-stranded RNA genome approximately 7.5 kb in length [7]. HAV has a single antigenic serotype that provides lifelong immunity after natural infection and follow the use of an inactivated vaccine. Despite the lack of antigenic variability, there are six viral genotypes of HAV (I–VI); three of the genotypes (I–III) are associated with human infections, and three (IV–VI) are associated with simian infections [8]. All the human genotypes (I–III) are divided into two subgenotypes (A and B), which exhibit a nucleotide variation of approximately 7.5% [9]. Recently, a new subgenotype IC has been proposed [10].

HAV transmission generally occurs through the fecal-oral route by ingestion of contaminated food, water, or through person-to-person contact. Person-to-person transmission is facilitated by close indoor contact where inadequate hygiene practices and a high proportion of susceptible individuals may exist. In recent years, many studies of indoor hepatitis A outbreaks have been published globally, but these studies are limited to outbreak investigations in daycare centers, nurseries, or schools [11], [12], [13]. Daycare centers and schools play an important role in the transmission network of HAV because of the tendency of children less than 6 years of age to develop asymptomatic infection and spread the disease among susceptible contacts [14]. However, our literature review identified few published reports investigating HAV transmission in the household environment, a favorable location for the exposure of susceptible individuals to infected individuals [15], [16], [17]. Within the domiciliary environment, the virus can spread through intimate contact between family members as seen for several pathogens [18], [19]. This study aimed to investigate the occurrence of the HAV transmission in the household environment.

## Materials and Methods

### Ethics Statement

This study was approved by the FIOCRUZ Ethics Committee (number: 135.261). All the subjects participating in the study or the responsible parent of the under the age subjects signed a consent form after being provided all the necessary and sufficient information to make an informed decision.

### Study population and sample collection

The study was conducted in the Viral Hepatitis Ambulatory of the Oswaldo Cruz Institute/Oswaldo Cruz Foundation (FIOCRUZ), Rio de Janeiro, Brazil, from November 2012 to April 2013. Patients who tested positive for anti-HAV IgM were invited to participate. Blood samples were collected from hepatitis A patients (index cases) and their family members (household contacts). Five milliliters of total blood from each individual was obtained by venipuncture, collected into sterile tube, and centrifuged. The serum was separated and stored at −20°C.

### Demographics, clinical, and laboratory data collection

For the epidemiologic evaluation, the following variables were obtained from the medical records of the index cases and their household contacts: age, sex, relationship between index cases and household contacts, presence or absence of symptoms, alanine aminotransferase (ALT), and aspartate aminotransferase (AST) serum levels.

### Household outbreak definition

For this study, a household outbreak was defined as the occurrence of two or more cases of hepatitis A in a single domiciliary environment. Thus, the presence of IgM anti-HAV and/or HAV RNA detected in the index case and in at least one of household contacts was characterized as a household outbreak.

### Serological tests for household samples

The serum samples for all household contacts were analyzed for specific antibodies against HAV (IgM and total anti-HAV) using an enzyme linked immunosorbent test (ETI-IGMK PLUS and ETI-AB-HAVK PLUS, DiaSorin, Italy). The results were recorded as positive or negative according to the standard procedures recommended by the manufacturer.

### Detection of HAV RNA in serum samples

Regardless of the serological test results, qualitative PCR and real-time PCR were performed on all the collected serum samples. The viral RNA was extracted from 140 µL of serum using the QIAmp Viral RNA Kit (Qiagen, Valencia, Spain). Reverse transcription was performed at 55°C for 1 h using 10 µl of RNA, random primers (Invitrogen, Rockville, MD, USA), and SuperScript III reverse transcriptase (Invitrogen). The VP1/2A region was amplified using a nested PCR as described elsewhere [11]. The PCR products were loaded onto a 1.5% agarose gel and stained with ethidium bromide to visualize the bands of an expected length of 247 bp. HAV RNA was quantified from 5 mL of cDNA using the TaqMan Real-time PCR assay (Applied Biosystems, Foster City, CA, USA). A specific synthetic ultramer (5' TTCGTGAGAGCCCTGGAAGAAAGAAGACGTATCAGAAAGCGTGAAAAATGAGTATGCGT GATTTAAGAACCCTGAACCTGCAGCTGATA3′) was used for the standard curve construction, and specific primers for the 5′ HAV non-coding region and a single-labeled 5′ FAM probe were used as described previously [20].

### Sequencing and phylogenetic analysis

Amplicons were purified using the QIAquick Gel extraction kit (Qiagen, Valencia, Spain) according to the manufacturer's recommendations. The nucleotide sequencing reaction was performed in both directions for HAV RNA positive samples with a Big Dye Terminator kit (Applied Biosystems, Foster City, CA, USA) and an automatic DNA sequencer (ABI Prism 3730, Applied Biosystems). The sequences obtained were edited using the Bioedit program and deposited in Genbank with access numbers between KF357524 and KF357561. The phylogenetic tree was constructed using the maximum-likelihood method and the Tamura-3-parameter with gamma distribution incorporating invariable sites (T92 +G+I) in the MEGA software package with 2000 replicates. The calculation of the nucleotide identity between the isolates from the index cases and their household contacts was also performed using the MEGA software package.

### Statistical Analysis

The data are expressed as frequencies, means ± standard deviations (SD), or medians and ranges (if quantitative variables were not normally distributed). In the bivariate analysis, we used the Chi-square (χ^2^) test for independence with Yate's continuity correction to compare proportions and nonparametric (Mann-Whitney *U* test) statistics to compare medians. For continuous variables with normal distributions (Kolmogorov-Smirnov test), homoscedasticity was tested by the Levene's test, and the unpaired Student's *t*-test was used to compare the means. The kappa (*k*) statistic was used to calculate the rate of agreement between the results of the nested PCR and real-time PCR. Based on the strength of the agreement, the *k* value was interpreted as follows http://www.sciencedirect.com/science/article/pii/S0264410X12011942- bib0080: <20%: poor; 21–40%: fair; 41–60%: moderate; 61–80%: good; and 81–100%: very good. A two-tailed *p* value<0.05 was considered statistically significant. Statistical analysis was performed using MedCalC for Windows, version 8.1.0.0 (MedCalc Software, Mariakerke, Belgium).

## Results

### Demographic, clinical, and laboratory data

A total of 97 patients from 30 families were included; 44 (45.4%) of the patients were male, and 53 (54.6%) were female. This population consisted of 30 index cases and 67 household contacts, ranging from 1–4 household contacts per index case. Frequencies of 1, 2, 3, and 4 household contacts per index case were observed in 8 (26.7%), 10 (33.3%), 9 (30.0%), and 3 (10.0%) cases, respectively. Approximately 48.4% (47/97) of the subjects were symptomatic or reported symptoms consistent with acute hepatitis, such as fever, nausea, vomiting, jaundice, and dark urine. Among the index cases, 43.3% (13/30) of the subjects were male, and 56.7% (17/30) were female, with a median age of 14.0 years (4–43.0 years); all patients had symptoms consistent with acute hepatitis. The median ALT and AST values of index cases were 186.0 U/L (16.0 to 1,530.0 U/L) and 115.0 U/L (10.0 to 1,800.0 U/L), respectively. Among all the household contacts included in this study, 46.3% (31/67) of the subjects were male, and 53.7% (36/67) were female; the median age was 27.0 years (1.0 to 67.0 years), and 25.4% (17/67) were symptomatic (Table 1).

**Table 1:** Demographic, clinic and laboratory data.

Data	Index case (n = 30)	Household contacts (n = 67)	Population (n = 97)
Gender, *n (%)*:			
Male	13 (43.3)	31 (46.3)	44 (45.4)
Female	17 (56.7)	36 (53.7)	53 (54.6)
Age (years):			
Median (range)	14.0 (4.0–43.0)	27.0 (1.0–67.0)	17.0 (1.0–67.0)
ALT (U/L):			
Median (range)	186.0 (16.0–1,530.0)	23.0 (4.0–1,840.0)	37.0 (4.0–1,840.0)
AST(U/L)			
Median (range)	115.0 (10.0–1,800.0)	35.0 (15.0–1,540.0)	4.0 (10.0–1,800.0).
Clinic, *n (%)*:			
Symptomatic	30 (100.0)	17 (25.4)	47 (48.4)
Asymptomatic	0 (0.0)	50 (74.6)	50 (51.6)

### Serological status of the household contacts

Hepatitis A cases were confirmed in 25.4% (17/67) of the household contacts after testing positive for IgM anti-HAV. In the household contacts, 67.2% (45/67) were positive for total anti-HAV antibodies, and 32.8% (22/67) were negative. Among the household contacts infected by HAV, 14 had symptoms, and three were asymptomatic.

### Detection of HAV RNA in serum samples

All samples included in this study (n = 97) were tested by qualitative nested PCR and quantitative real-time PCR. HAV RNA was detected by qualitative PCR in 40.2% (39/97) of samples and by real-time PCR in 48.4% (47/97). The rate of agreement (Kappa index) between the qualitative PCR and real-time PCR results was 79.3%, indicating good agreement between these methods.

Analyzing only the index cases (n = 30), HAV RNA was detected in 80.0% (24/30) and 93.3% (28/30) of the serum samples by qualitative-PCR and real-time PCR, respectively. The median viral load of the index case samples was 10^3^ copies/ml (10^2^–10^6^ copies/ml). Among the household contacts (n = 67), HAV RNA was detected in 22.4% (15/67) of the serum samples by nested PCR. Of the fifteen samples testing positive by nested PCR, 12 were IgM anti-HAV-positive, two were positive for total anti-HAV antibodies, and one was negative for total anti-HAV antibodies.

HAV RNA was detected in 19 household contacts (28.4%) by real-time PCR. The median viral load of the household contact samples was 10^3^ copies/ml (10^2^–10^5^ copies/ml). Fourteen household samples had HAV RNA simultaneously detected by nested PCR and real-time PCR, whereas five samples had HAV RNA detected only by real-time PCR. Of these five samples, three showed reactivity for total anti-HAV antibodies, and two were reactive for only IgM anti-HAV. One sample from a household contact that tested positive by nested PCR tested negative by real-time PCR.

Using molecular biology tools (nested PCR and real-time PCR), it was possible to detect HAV RNA in 20 household contacts. The number of infected household contacts detected by molecular biology was slightly higher than that found by serology (17 household contacts infected).

### Detection of household outbreaks and analysis of factors associated with HAV infection

To determine the number of the household outbreaks of hepatitis A in this study, the serology and molecular biology results were analyzed together; not all individuals reactive for IgM anti-HAV had HAV RNA detected, and not all individuals with HAV RNA detected in the serum sample were reactive for IgM anti-HAV. Altogether, sixteen household outbreaks were detected, involving 16 index cases and 23 household contacts. Compared with analyzing the results separately, the combined analysis of the serology and molecular biology results gave a higher number of household contacts affected by HAV.

In the bivariate analysis, age and aminotransferase levels were associated with hepatitis A status (infected *vs.* non-infected) among the household contacts (Table 2).

**Table 2:** Analysis of factors related to HAV infection among household contacts (n = 67).

	Hepatitis A		
	Presence (*n* = 23)	Absence (*n* = 44)	*P*- value
Age (years):			
Mean ± SD	17.4±14.4	31.5±18.2	**<0.0001** †
Gender, *n* (%):			
Male	13 (56.5)	18 (40.9)	0.3376
Female	10 (43.5)	26 (59.1)	
ALT (U/L):			
Median (range)	38.0 (11.0–1,840.0)	22.0 (4.0–87.0)	**0.0041**
AST (U/L):			
Median (range)	44.0 (22.0–1,540.0)	33,0 (15,0–64,0)	**0.0036**
Kinship-index case, *n* (%):			
Father/Mother	10 (43.5)	22 (50.0)	0.7803
Siblings	7 (30.4)	10 (22.7)	
Other*	6 (26.1)	12 (27.3)	

*Grandfather, uncle, sister-in-law, boyfriend and others.

† Infected *versus* noninfected (susceptible and immune individuals). SD, standard deviation.

### Molecular characterization of HAV isolates and investigation of HAV household transmission

**Figure 1:**
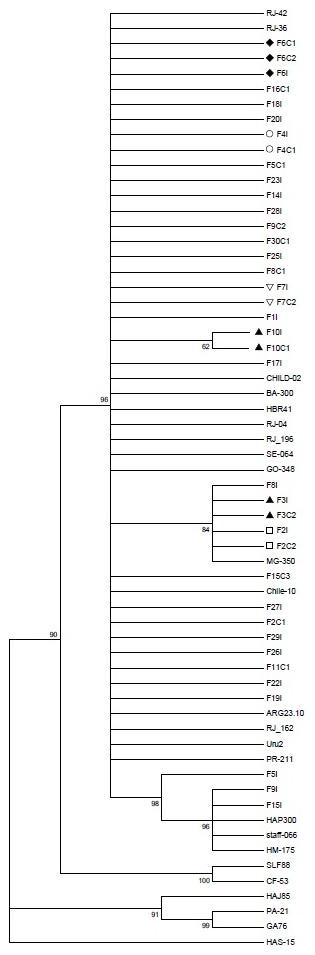
Phylogenetic analysis of 39 HAV isolates based on 168 nucleotides of VP1/2A (position 3024–3191), constructed by the method of Maximum Likelihood. The value of bootstrap was calculated from 2,000 replicates. Strains marked by symbols refer to families who had isolates with high nucleotide identity.

All 39 HAV isolates obtained belonged to the genotype I; 36 belonged to subgenotype IA and three to subgenotype IB (Figure 1). The strains from different household outbreaks showed a nucleotide similarity ranging from 87.4–100%. The sequencing analysis from the index cases and their household contacts was obtained for 10 families; in six families, the circulating strains within the household environment had a high nucleotide identity (100%), suggesting that the strains infecting the index case and the corresponding household contact were the same (Table 3).

**Table 3:** Nucleotide similarity matrix between HAV isolates obtained in serum samples from individuals involved in the household outbreaks.

	F2I	F2C2	F2C1	F3I	F3C2	F4I	F4C1	F5I	F5C1	F6I	F6C1	F6C2	F7I	F7C2	F8I	F8C1	F9I	F9C2	F10I	F10C1	F15I	F15C3
F2I																						
**F2C2**	**0,000**																					
F2C1	0,037	0,037																				
F3I	0,006	0,006	0,031																			
**F3C2**	0,006	0,006	0,031	**0,000**																		
F4I	0,031	0,031	0,044	0,025	0,025																	
**F4C1**	0,031	0,031	0,044	0,025	0,025	**0,000**																
F5I	0,090	0,090	0,077	0,097	0,097	0,112	0,112															
F5C1	0,031	0,031	0,044	0,025	0,025	0,000	0,000	0,112														
F6I	0,031	0,031	0,044	0,025	0,025	0,000	0,000	0,112	0,000													
**F6C1**	0,031	0,031	0,044	0,025	0,025	0,000	0,000	0,112	0,000	**0,000**												
**F6C2**	0,031	0,031	0,044	0,025	0,025	0,000	0,000	0,112	0,000	**0,000**	0,000											
F7I	0,031	0,031	0,044	0,025	0,025	0,000	0,000	0,112	0,000	0,000	0,000	0,000										
**F7C2**	0,031	0,031	0,044	0,025	0,025	0,000	0,000	0,112	0,000	0,000	0,000	0,000	**0,000**									
F8I	0,000	0,000	0,037	0,006	0,006	0,031	0,031	0,090	0,031	0,031	0,031	0,031	0,031	0,031								
F8C1	0,031	0,031	0,044	0,025	0,025	0,000	0,000	0,112	0,000	0,000	0,000	0,000	0,000	0,000	0,031							
F9I	0,089	0,089	0,089	0,096	0,096	0,126	0,126	0,024	0,126	0,126	0,126	0,126	0,126	0,126	0,089	0,126						
F9C2	0,031	0,031	0,044	0,025	0,025	0,000	0,000	0,112	0,000	0,000	0,000	0,000	0,000	0,000	0,031	0,000	0,126					
F10I	0,037	0,037	0,037	0,031	0,031	0,006	0,006	0,105	0,006	0,006	0,006	0,006	0,006	0,006	0,037	0,006	0,118	0,006				
**F10C1**	0,037	0,037	0,037	0,031	0,031	0,006	0,006	0,105	0,006	0,006	0,006	0,006	0,006	0,006	0,037	0,006	0,118	0,006	**0,000**			
F15I	0,089	0,089	0,089	0,096	0,096	0,126	0,126	0,024	0,126	0,126	0,126	0,126	0,126	0,126	0,089	0,126	0,000	0,126	0,118	0,118		
F15C3	0,031	0,031	0,043	0,024	0,024	0,037	0,037	0,097	0,037	0,037	0,037	0,037	0,037	0,037	0,031	0,037	0,110	0,037	0,044	0,044	0,110	

## Discussion

Hepatitis A is an endemic disease in developing countries that primarily affects children and is transmitted through the fecal-oral route. The probability of fecal-oral transmission increases with extended and close personal contact between infected and susceptible individuals. The prolonged shedding of HAV before and after the onset of symptoms, in association with the lack of good hygienic practices and the sharing of objects in the domiciliary environment, may contribute to a more suitable scenario for person-to-person HAV transmission, as previously reported for several diseases with fecal-oral transmission [18], [19], [21]. Although person-to-person transmission is common in hepatitis A, few studies have evidenced this mode of transmission [16], [17]. In this study, index cases and contacts were investigated to detect possible hepatitis A household outbreaks. Clinical and serological investigations were conducted among the household contacts of the index cases. Seventeen household contacts were IgM anti-HAV-positive, 22 were susceptive to HAV, and 28 exhibited immunity to HAV. Despite having found whole families with positive serology for HAV infection, these data are not sufficient to confirm the household transmission of HAV in these outbreaks. For this reason, we performed the detection, quantification, and sequencing of HAV RNA to establish the epidemiological relationships between the isolates. HAV RNA was detected by nested PCR in 40.2% (39/97) of the samples, and real-time PCR was able to detect HAV RNA in 48.4% (47/97) of the samples. Real-time PCR had a higher sensitivity than nested PCR [20], [22], and thus, samples with a low viral load were negative by nested PCR.

HAV RNA was also investigated in the household contacts that were seronegative or had previous immunity to hepatitis A; one anti-HAV-negative and five IgG anti-HAV-positive household contacts had HAV RNA detected in their serum samples. These findings demonstrate the importance of molecular biology techniques for the early diagnosis of HAV infection during the viral incubation phase and for the detection of the virus for longer periods than are commonly described [23], [24]. With HAV RNA detection, the number of infected household contacts increased from 17 to 23, and the number of household outbreaks detected also increased from 13 to 16.

In this study, a significant difference between the mean age of the contacts with and without hepatitis A was observed. Younger household contacts had a higher frequency of HAV infection, corroborating recent studies showing an increase in the age at which HAV infection occurs [4], [25], [26]. However, the presence of previous immunity was associated with older (>20 years of age) household contacts; this finding was in accordance with many epidemiological studies reporting a strong association between age and anti-HAV prevalence [5], [26], [27], [28]. Phylogenetic analysis of HAV isolates obtained from household outbreaks revealed a co-circulation of the subgenotypes IA and IB. Several studies have reported the co-circulation of subgenotypes IA and IB in Brazil [11], [12], [29]. In 10 families, it was possible to sequence HAV RNA from the index cases and their household contacts. When a similarity nucleotide matrix was performed, 100% homology between the index case and contact strains was observed in six of the families, suggesting that person-to-person transmission may have occurred. However, in three families, the index cases and their household contacts were infected by different subgenotypes of HAV, suggesting different sources of infection. Wu et al., 2014 showed through ultra-deep pyrosequences (UDPSs) of HAV 5**′**-untranslated region (5**′**UTR) that minor nucleotide substitutions of this region can occur in HAV strains derived from a single source outbreak, and that these nucleotide substitutions were different from those of the sporadic case. So, UDPS analysis might be a new analytical tool for the source of hepatitis A outbreaks [30].Person-to-person transmission is the predominant mode of HAV spread when there is prolonged and intimate contact between susceptible individuals and those with HAV infection. One of the main risk factors for acquiring this infection is family life [31], especially for children without proper hygienic habits. In the six families in which person-to-person transmission was suggested, four presented children as index cases (4–7 years), reinforcing that the person-to-person transmission might have occurred.

Although the person-to-person transmission of HAV has been described for indoor environments, there is no current work in Brazil using nucleotide similarity between the strains to provide evidence for such transmission in the household environment. Person-to-person transmission has been suggested by molecular epidemiology studies of household outbreaks caused by other fecal-oral transmission agents, such as rotavirus and enterovirus 71; these studies have reported a nucleotide identity of over 99% among strains circulating in symptomatic family members [18], [32]

Indoor hepatitis A outbreaks represent a public health problem, requiring the investigation of the etiology and rapid action measures to control the infection. The results of this study demonstrate the relevance of HAV transmission in the household environment and support the need for the implementation of the hepatitis A vaccine not only in children but also in susceptible household contacts as a means of preventing the spread of the disease and reducing the disease impact on susceptible individuals in contact with infected persons.
